# The spatio-functional cortical representation of muscles innervated by the accessory nerve using nTMS

**DOI:** 10.1016/j.cnp.2025.11.004

**Published:** 2025-11-26

**Authors:** J. Reinsch, M. Denker, M. Engelhardt, A. Zdunczyk, D. Huscher, P. Vajkoczy, T. Picht, N.F. Dengler

**Affiliations:** a Charité – Universitätsmedizin Berlin, Corporate Member of Freie Universität Berlin and Humboldt-Universität zu Berlin, Klinik für Neurochirurgie, Charitéplatz 1, 10117 Berlin, Germany; b Charité – Universitätsmedizin Berlin, Corporate Member of Freie Universität Berlin and Humboldt-Universität zu Berlin, Image Guidance Lab, Charitéplatz 1, 10117 Berlin, Germany; c Department of Neurosurgery, Vivantes Klinikum im Friedrichshain, Landsberger Allee 49, 10249 Berlin, Germany; d Charité – Universitätsmedizin Berlin, Corporate Member of Freie Universität Berlin and Humboldt-Universität zu Berlin, Department of Biometry and Clinical Epidemiology, Charitéplatz 1, 10117 Berlin, Germany; e Faculty of Health Sciences Brandenburg, Medical School Theodor Fontane, Campus Bad Saarow, Germany; f Department of Neurosurgery, HELIOS Hospital Bad Saarow, Germany

**Keywords:** nTMS, Accessory nerve, Cortical muscle representation, Sternocleidomastoid muscle, Trapezius muscle

## Abstract

**Objective:**

The accessory nerve is frequently affected by injury which causes disability due to a loss of function in the trapezius muscle and the sternocleidomastoid muscle. Navigated transcranial magnetic stimulation (nTMS) may be a valuable tool to quantify aspects of cortical motor reorganization.

**Methods:**

nTMS was used to stimulate the motor cortex in 15 healthy volunteers and 3 accessory nerve lesion patients to determine the representation of the sternocleidomastoid muscle, trapezius, deltoid, biceps, first dorsal interosseus and zygomatic major muscles.

**Results:**

The center of gravitiy (CoG) of the sternocleidomastoid muscle was located between the face (zygomatic major muscle) and hand (first dorsal interosseus muscle). CoG of the trapezius muscle was located superiorly to the hand. Distances to the lateral sulcus differed significantly (median: trapezius muscle 54.9 [9.4] mm, sternocleidomastoid muscle 30.1 [15.7] mm, p < 0.001). The radius (in mm) of the area of the sternocleidomastoid muscle was significantly larger compared to the trapezius (14.3 [5.7] and 11.5 [6.9], p = 0.004).

**Conclusion:**

This study describes the detailed spatio-functional representation of trapezius and sternocleidomastoid muscle in comparison to four other muscles.

**Significance:**

Our data may serve as a baseline for future studies on patients with accessory nerve injuries and a decision-making tool for the indication and timing for reconstructive surgery.

## Highlights

• nTMS mapping of cortical muscle areas in accessory nerve lesions vs. controls.
• Sternocleidomastoideus and trapezius differed in cortical site, size and laterality.
• Motor thresholds higher contralateral; SCM area smaller or absent post-transection.

## 1 Introduction

The accessory nerve innervates both the trapezius and sternocleidomastoid muscle. It is the most frequently iatrogenically injured cranial nerve, which causes considerable disability and pain and therefore affects quality of life (Goransson et al., 2016, Rasulic et al., 2017, Sakamoto et al., 2021).

While the functions of the trapezius muscle include elevation of the arm over 90 degrees and stabilizing the shoulder, the sternocleidomastoid muscle rotates and inclines the head and serves as a respiratory muscle when the head is fixed.

Many patients with an accessory nerve injury already benefit from surgical nerve repair (Matz and Barbaro, 1996). To improve outcomes, knowledge about the influence of cortical plasticity of the motor areas of these two muscles provides a basis for decision making (Anastakis et al., 2008, Schnupp and Kacelnik, 2002, Socolovsky et al., 2017).

Markers to predict which patients benefit from a surgical intervention compared to a conservative strategy as well as the timing of a surgical repair are currently lacking. In this study we investigated whether navigated transcranial magnetic stimulation (nTMS) can identify such markers by providing a detailed assessment of the brain areas responsible for these two muscles (Fu and Gordon, 1995, Kraus and Gharabaghi, 2016, Xerri, 2012). The aim of our study was to identify the localization of the trapezius and sternocleidomastoid muscle on the precentral gyrus on the motor cortex using nTMS and calculate other parameters on healthy subjects that could change in patients with accessory nerve lesions.

Previous studies have shown contradicting evidence for the location of the trapezius and sternocleidomastoid muscle in healthy subjects. Some studies consider the sternocleidomastoid muscle being represented near the location of the face on the motor cortex (Penfield W, 1950). Some localized it close to the trunk region, which is between the arm and leg area on the precentral gyrus (Kang et al., 2011, Thompson et al., 1997). This study lies a focus on detecting the exact cortical location of both of these muscles to serve as a base for detecting a possible shift in patients.

In terms of neuronal connection of the accessory nerve, there is some evidence of bilateral control of both trapezius and sternocleidomastoideus, which presumably happens at the level of the brainstem. One study observed bilateral MEPs (motor evoked potentials) after a magnetic stimulation of the trapezius on one hemisphere (Alexander et al., 2007). There is also evidence for interhemispheric connections for trapezius in terms of interhemispheric inhibition (Ginatempo et al., 2019, Matthews et al., 2013). For sternocleidomastoideus, bilateral responses can also be found after unilateral stimulation, although the ipsilateral responses need higher stimulation intensities and have a longer latency (Benecke et al., 1988, Gandevia and Applegate, 1988). Other studies found ipsi- or bilateral responses depending on the localization of the stimulation or MEPs being mostly contralateral, but partly also bilateral (Benecke and Meyer, 1991, Berardelli et al., 1991). There are hypotheses that the sternocleidomastoid muscle could even be controlled mostly by the ipsilateral hemisphere (Mazzini and Schieppati, 1992, Odergren and Rimpilainen, 1996). Thus, the present study further aimed to study these bilateral effects by recording MEPs from both sides of the body.

nTMS is a valuable tool for the painless and non-invasive mapping of the cortical organization of the somatotopy according to the homunculus (Picht, 2014, Rossini et al., 2015, Schmidt et al., 2015).

This study provides an nTMS mapping protocol from both hemispheres for the sternocleidomastoid muscle, trapezius, deltoid, biceps, first dorsal interosseus and zygomatic major muscles that measures the location on the cortex, the excitability and inhibition of the respective areas (in detail: the resting motor threshold (RMT), latency and amplitude, the center of gravity (CoG), motor area (area), the recruitment curve (RC) and the cortical silent period (CSP)). The reason for mapping the muscles of the hand (first dorsal interosseus muscle) and arm/shoulder (biceps and deltoid muscle) is to have a reliable, often-used muscle in nTMS (first dorsal interosseus muscle) and to map two muscles which are both anatomically and functionally close to trapezius and sternocleidomastoid muscle (biceps and deltoid muscle) (Boroojerdi et al., 1999). This completes with the zygomatic major, which represents the cortical location of the face.

To complete this study, data from three patients with an accessory nerve lesion partly before or after reparative surgery (patient 1: TMS-mapping 24 months after surgery, patient 2: lesioned eight months before TMS mapping, patient 3: sustained a lesion seven months before the TMS mapping) has been collected, which could serve as a baseline for future clinical studies.

## 2 Methods

### 2.1 Study design

Fifteen healthy subjects (4 male, 11 female; mean age: 26,9 yr; range: 21–33 yr) as well as three patients with accessory nerve injury (1 male, 2 female; mean age: 49,3 yr; range: 39–61 yr, all with right-sided lesions) participated in the study (Table 1). Participants were asked about their handedness (subjects: 14 right-handed, 1 left-handed; patients: all right-handed). The inclusion criteria were 18 years of age or older, no contraindications for TMS or MRI and the ability to provide written informed consent. Exclusion criteria were a neurological or psychiatric illness apart from the accessory nerve lesion for the patient group. All study procedures were approved by the local ethics committee (EA4/033/21). The study was conducted in accordance with the Declaration of Helsinki. All subjects provided their written informed consent.

**Table 1:** Characteristics of the 15 healthy subjects are displayed, which includes the age (mean: 26.9; range: 21–33), the sex (73.3% female), and the handedness (93.3% right). Underneath, the same characteristics, including the side of the lesion (100% right), for the patients are displayed. In addition, a short summary of the patients’ history and treatment is given.

**Subject**	**Age**	**Sex**	**Handedness**	**Lesioned side**
1	26	f	r	−
2	29	f	r	−
3	23	m	r	−
4	27	f	l	−
5	28	f	r	−
6	27	f	r	−
7	33	m	r	−
8	32	m	r	−
9	23	f	r	−
10	21	f	r	−
11	33	f	r	−
12	30	f	r	−
13	24	m	r	−
14	26	f	r	−
15	22	f	r	−
			
pat 1	61	m	r	r
	- was mapped 24 months after surgery (reconstruction with suralis nerve graft) - still presented with a paralyzed trap on the right side after a neck dissection - scm and trap could not be determined for the lesioned side, although an EMG follow-up showed nerve regeneration postop			
pat 2	39	f	r	r
	- sustained a lesion on the right accessory nerve during a lymph node biopsy 8 months before the TMS mapping (preop) - an EMG showed no signs of reinnervation of the accessory nerve - the scm showed no signs of motor deficits, the patient had a winged scapula and a shoulder depression on the right side - intraoperative finding: complete transection of the accessory nerve			
pat 3	48	f	r	r
	- sustained a lesion on the right accessory nerve during a lymph node biopsy 7 months before the TMS mapping (preop) - also presented with an atrophic trap on the right side - intraoperative finding: nerve intact, compressed in scar tissue			

Characteristics of the subjects and patients included in the study.

*Abbreviations*: f – female; m – male; r – right; l – left; pat – patient.

### 2.2 MRI image acquisition

Participants brought their own structural MRI scan (MPRAGE sequence) with them. The patients had been examined at a 3 T MRI (Magnetom Vida, Siemens, Erlangen, Germany) equipped with a 20-channel receiver head coil. The MRI protocol included a T1-weighted anatomical MPRAGE MRI scan (isotropic resolution 1 mm; TR/TE 2300/2.3 ms; TI 900 ms; flip angle 8 degrees; 192 slices) and a DTI sequence (70 slices with a slice thickness of 2.0 mm, TR/TE 8100/75 ms, 5 b0 volumes and 2 averages of 20 directions with a b-value of 1000 s/mm2).

### 2.3 EMG and Neuronavigated TMS

nTMS was applied using a Nexstim NBS5 stimulator (Nexstim, Helsinki, Finland) with a figure of eight coil with opposite current directions (outer diameter 70 mm) by orienting the coil perpendicular to major sulci in the region of the area of the targeted gyri and applying single pulses (exception: repetitive pulses for the recruitment curve). Rotation and angulation were varied a bit to find a hotspot. Each participant’s MRI was uploaded as a subject-specific navigational dataset for the nTMS assessment. MEPs were recorded first for the right, then for the left hemisphere for all muscles. For the sternocleidomastoid muscle (scm) and trapezius (trap), EMG was always active for the contra- and ipsilateral muscle to detect possible bilateral responses. Disposable Ag/AgCl surface electrodes (Neuroline 700, Ambu, Ballerup, Denmark) were used for EMG recording on the tendon and belly of each muscle. In detail, electrodes were placed on each muscle as follows: For scm, the muscle electrode had been placed on the middle part of the medial head, whereas the tendon electrode had been placed on the tendon to manubrium sterni. For trap, the muscle electrode had been placed on the middle part of the descending part of the muscle, the tendon electrode on the tendon which is attached to the lateral clavicle. For the deltoid muscle (del), the tendon to the humerus has been used. For biceps (bbm), we used the distal tendon to the radius. For the first dorsal interosseus (fdi), we used the distal tendon to the proximal phalanx and for zygomaticus (zyg), the tendon electrode had been placed over the tendon to the zygomatic arch. The ground electrode had been placed on the wrist on the ulnar head and participants were told to sit relaxed and comfortably.

### 2.4 Parameters and mapping protocol

Each muscle was mapped separately. Based on literature only muscle responses with a latency of 5–20 ms and amplitude > 50uV were counted (Alexander et al., 2007, Menon et al., 2018). At first, a hotspot was determined as the point, rotation and angulation consistently producing the largest response. Third, the RMT was determined as the lowest stimulation intensity to produce MEPs larger than 50 µV in five out of ten attempts (Rossini et al., 2015). Thereafter, an area mapping at 105 % RMT was performed. A circle of stimulations resulting in no responses had been created around the hotspot and later “filled” with stimulations that ideally increased in amplitude towards the center of the circle. This was used later for the calculation for the CoG. After this, a RC was determined using 70 to 80 stimuli with varying intensities with an intensity between 80–130 or 140 % of the RMT respectively. MEP amplitudes were recorded as outcomes. Lastly, CSP was determined for all muscles except the scm. Here, subjects were told to contract the respective muscle, while 10 stimuli with an intensity of 130 % RMT were given over the muscle hotspot.

### 2.5 Data processing and statistical analysis

After the measurement, EMG responses were manually checked to exclude false responses due to accidental movements, tension of the muscle or electrodes not adhering anymore, e.g., because of sweat. Latencies (in ms) and amplitudes (in μV) of the MEPs were measured manually and calculated as the mean of all values from stimulations with the determined RMT over the hotspot for the respective muscle and hemisphere. CoG, area and radius were graphically determined using the Convex Hull Method in a custom-written Matlab script (MATLAB R2022a, Mathworks, Natick, Massachusetts, United States). The CoG represents the location most likely to produce the largest MEP considering all other given responses of the muscle (Sondergaard et al., 2021). It is considered as more exact than the hotspot. The size of the area (in mm^2^) and the concerning radius (in mm) were also calculated. From the RC, the slope, V50 and area under the curve were determined using Prism (Prism 9, GraphPad Software, Inc., Boston, Massachusetts, United States). For that, MEP amplitudes were plotted against the stimulation intensities (expressed in % of the RMT) and a Boltzmann sigmoidal function was fit to the data (Kukke et al., 2014). The distance to the lateral sulcus was determined by calculating the smallest distance between the CoG of a muscle and the point on the lateral sulcus closest to the central sulcus at a peeling depth of 22.5 mm. Therefore, we marked the respective point as well as the lateral sulcus on the MRI and determined the shortest possible distance between them. The CSP had been determined manually by measuring the duration of the silent part of the EMG after a stimulus with an intensity of 130 % RMT and by calculating the mean of 10 stimulations per muscle.

The statistical analysis was conducted in SPSS (IBM SPSS Statistics 27, Chicago, Illinois, United States). First, the data was checked for normal distribution using the Shapiro-Wilk test and descriptive statistics were calculated. In addition, P-P- and Q-Q-plots were also checked subjectively. Thereafter, a comparison between each muscle on the non-dominant side (nds) and the corresponding one on the dominant side (ds) (e.g., scm (ds) – scm (nds), as well as between all the six different muscles regardless the side (e.g., scm – trap) was made. For normally distributed data, the *t*-test for paired samples was used. Otherwise, the Wilcoxon test was chosen. Results counted as significant if p < 0.05.

## 3 Results

### 3.1 Feasibility of nTMS for muscles innervated by the accessory nerve

All subjects generally tolerated the study procedures well and without side effects. The duration of a single session per subject was between 3:47 h and 5:00 h (median: 4:18 [0:27] h). For scm, stimulation over the area of the temporal muscle had been uncomfortable to some subjects. Seven subjects and all patients described the procedure as partly painful. Most of the subjects did not tolerate the mapping of the scm CSP because of the high intensities. Furthermore, it was difficult for the examiner to reach the most rostral regions during hotspot determination and area mapping of scm. Only two subjects were able to keep the scm in a sufficiently tensed condition, what lead to the cancellation of the mapping of the scm CSP. For trap, the EMG signal had to be manually inspected for cardiac activity due to the close position of the trap electrodes to the heart.

### 3.2 Cortical representation of sternocleidomastoid and trapezius muscle

**Fig. 1:**
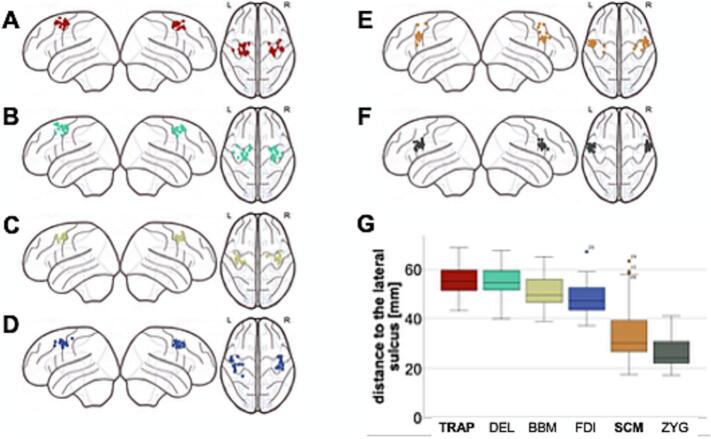
Representation of the Center of Gravity of the six muscles that were measured (A: trapezius muscle (TRAP), B: deltoid muscle (DEL), C: biceps brachii muscle (BBM), D: first dorsal interosseus (FDI), E: sternocleidomastoid muscle (SCM), F: zygomatic major muscle (ZYG) and G: the distance to the lateral sulcus of the Centers of Gravity of those muscles mentioned above).

The CoG of the scm was located between the CoG of the face (zyg) and the hand (fdi). The CoG of the trap was located superiorly to the CoG of the hand and arm (biceps) and overlapped largely with the deltoid (Fig. 1). Distances to the lateral sulcus differed significantly between the trap and scm (median: trap 54.9 [9.4] mm, scm 30.1 [15.7] mm, p < 0.001). The radius of the area of the scm was significantly larger compared to the trap (14.3 [5.7] mm and 11.5 [6.9] mm, p = 0.004) and also compared to the other muscles (zyg 11.2 [5.8] mm; deltoid 11.2 [5.5] mm; bbm 11.2 [5.1] mm; fdi 10.4 [3.9] mm). The area of the trap differed significantly in size between the ds and nds (nds: 575.8 [386.3] mm^2^; ds: 264.0 [307.0] mm^2^, p = 0.04). Concomitantly, the radius of the trap also differed significantly between both hemispheres (nds:13.8 [4.8] mm; ds: 10.1 [5.1] mm, p = 0.04). In comparison to that, the area of scm did not differ between ds and nds.

### 3.3 Comparison of nTMS parameters for different muscles

**Fig. 2:**
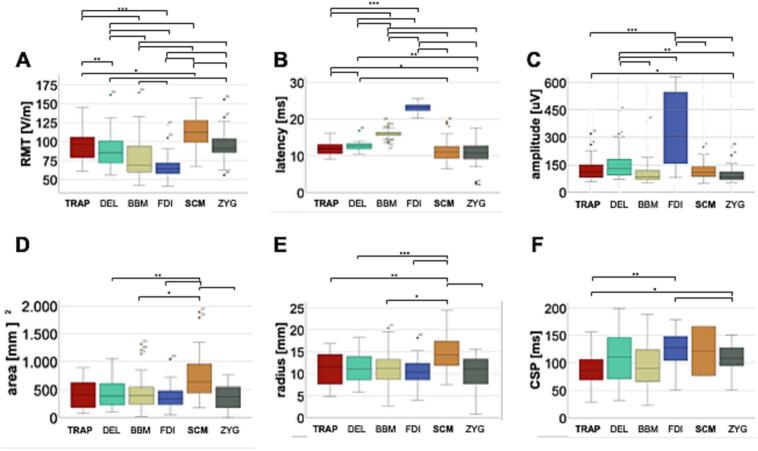
Figurative representation of A: the resting motor threshold (RMT), B: the latency, C: the amplitude, D: the area, E: the radius, and F: the cortical silent period (CSP) of the trapezius muscle (TRAP), deltoid muscle (DEL), biceps brachii muscle (BBM), first dorsal interosseus (FDI), sternocleidomastoid muscle (SCM), and zygomatic major muscle (ZYG) of the healthy volunteers.

The (hemisphere-independent) median RMT of the scm and trap were 112.0 [30.0] V/m and 96.0 [30.0] V/m respectively and therefore considerably higher than the mean RMT of the fdi (64.0 [14.0] V/m). The RMT of the scm was significantly higher than the RMT of the trap (p = 0.001, Fig. 2).

We observed significantly shorter latencies of the MEPs of the scm (median: 11.0 [3.9] ms) and trap (median: 11.9 [2.6] ms) compared to those of the bbm (median: 16.1 [1.1] ms; p = 0.001 for both) and the fdi (median: 23.2 [1.6] ms; p = 0.001 for both). Overall, the latency increased with the distance from the motor cortex to the location of the muscle. The amplitude of the MEPs of both scm (median: 108.0 [59.0] μV) and trap (median: 110.5 [72.0] μV) differed significantly from fdi (median: 302.5 [417.0] μV; p = 0.001 for both).

**Fig. 3:**
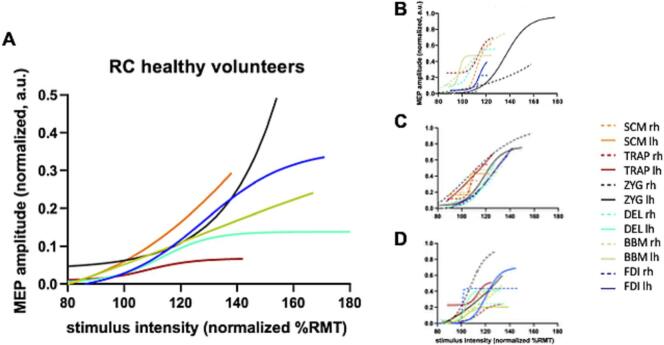
Figurative representation of the Recruitment Curves (RC) of A: the healthy subjects as well as B, C, and D: the lesioned patients 1, 2, and 3 (trapezius muscle (TRAP), deltoid muscle (DEL), biceps brachii muscle (BBM), first dorsal interosseus (FDI), sternocleidomastoid muscle (SCM), zygomatic major muscle (ZYG), left hemisphere (lh), right hemisphere (rh), motor evoked potentials (MEP), resting motor threshold (RMT).

For the RC, V50, slope and area under the curve had been calculated. For V50, scm had a median of 109.7 [18.5] %RMT and trap of 121.2 [86.8] %RMT. Trap was significantly higher than bbm (median: 109.2 [17.0] %RMT; p = 0.018). The median slope of scm and trap were 8.1 [22.1] and 6.6 [18.6] and didn’t differ from the other muscles significantly. The area under the curve was 9.9 [6.9] for scm and 7.6 [6.6] for trap, which is significantly lower than the area under the curve for the bbm (14.0 [11.0]; p = 0.025 and p = 0.001) and the fdi (16.4 [15.5]; p = 0.001 for both). Furthermore, the trap differs significantly from zyg (median: 12.5 [7.6]; p = 0.005, Fig. 3).

For trap, the CSP was significantly shorter (median: 86.0 [39.0] ms) than in zyg (median: 108.0 [34.0]; p = 0.031) and fdi (median: 128.0 [46.0]; p = 0.001), respectively (Table 2).

**Table 2:** The muscle specific stimulation parameters of the 6 determined muscles in 15 healthy subjects are displayed. In detail, the median and the interquartile range [in square brackets] are displayed.

**Muscle**	**RMT [V/m]**	**Latency [ms]**	**Amplitude [μV]**	**Distance to lateral sulcus [mm]**	**Area [mm^2^]**	**Radius [mm]**	**V50**	**Slope**	**Area under the curve**	**CSP [ms]**
**TRAP**	96.0 [30.0]	11.9 [2.6]	110.5 [72.0]	54.9 [9.4]	413.0 [458.0]	11.5 [6.9]	121.2 [86.8]	6.6 [18.6]	7.6 [6.6]	86.0 [39.0]
**DEL**	85.5 [30.0]	12.7 [1.4]	129.0 [92.0]	54.7 [8.6]	391.0 [388.3]	11.2 [5.5]	121.1 [115.0]	8.2 [29.4]	8.8 [6.0]	110.0 [78.5]
**BBM**	69.0 [37.0]	16.1 [1.1]	83.5 [48.8]	49.5 [9.8]	398.0 [357.5]	11.2 [5.1]	109.2 [17.0]	9.2 [10.3]	14.0 [11.0]	90.0 [70.0]
**FDI**	64.0 [14.0]	23.2 [1.6]	302.5 [417.0]	47.1 [9.5]	341.0 [255.8]	10.4 [3.9]	121.9 [16.7]	8.6 [4.3]	16.4 [15.5]	128.0 [46.0]
**SCM**	112.0 [30.0]	11.0 [3.9]	108.0 [59.0]	30.1 [15.7]	636.5 [531.0]	14.3 [5.7]	109.7 [18.5]	8.1 [22.1]	9.9 [6.9]	121.5
**ZYG**	91.0 [19.0]	10.8 [3.6]	83.0 [43.0]	24.3 [9.4]	373.4 [373.5]	11.2 [5.8]	115.5 [14.0]	10.8 [10.1]	12.5 [7.6]	108.0 [34.0]

Muscle specific stimulation parameters in healthy subjects.

*Abbreviations*: RMT – resting motor threshold; CSP – cortical silent period; SCM – sternocleidomastoid muscle; TRAP – trapezius muscle; ZYG – zygomaticus muscle; DEL – deltoid muscle; BBM – biceps muscle; FDI – first dorsal interosseus muscle.

### 3.4 Effect of hemispheric dominance

As for hemispheric differences, the RMT differed in the scm (ds: 112.0 [37.0] V/m; nds: 111.0 [32.0] V/m; p = 0.05), the deltoid (ds: 74.0 [23.0] V/m; nds: 91.0 [36.0] V/m; p = 0.02), the bbm (ds: 67.0 [17.0] V/m; nds: 74.0 [51.0] V/m; p = 0.04] and the fdi (ds: 61.0 [13.0] V/m; nds: 70.0 [13.0] V/m; p = 0.03). The latency of scm was higher on the ds (11.2 [5.1] ms) than on the nds (10.2 [3.0] ms, p = 0.01). The area of trap was larger on the nds compared to the ds (575.8 [386.3] mm^2^ compared to 264.0 [307.0] mm^2^; p = 0.04). Accordingly, the same holds true for the radius (trap: nds 13.8 [4.8] mm vs. ds 10.1 [5.1] mm; p = 0.04).

For the RC, a significant difference between the hemispheres could only be detected for the area under the curve, which was higher for the trap on the ds (nds 5.7 [5.6] vs. ds 9.6 [4.8]; p = 0.01).

Apart from this, there were no other significant differences (Table 4).

### 3.5 nTMS in patients with NXI lesion

Table 1, Table 3 depict clinical characteristics, pathologies and nTMS mapping results in three patients with NXI lesion. In summary, for these three exemplary patients, RMTs were found to be higher for the nds when the nerve of the ds was found transected. One patient had the interesting finding of a higher distance to the lateral sulcus of the scm, especially on the (transected) ds. For the (injured) ds, scm area was smaller or not detectable in the two patients with the completely transected nerve, whereas it was larger for the patient with the accessory nerve that was only compressed in scar tissue. For details, including RC measuring results, see (Table 3; Fig. 3).

**Table 3:** The muscle specific stimulation parameters of the dominant and nondominant side in patient 1,2, and 3 are displayed.

**Muscle**	S**ide**	**RMT** **[V/m]**			**Latency [ms]**			**Amplitude [μV]**			**Distance to the lateral sulcus [mm]**			**Area [mm^2^]**			**Radius [mm]**			**V50**			**Slope**			**Area under the curve**			**CSP [ms]**		
**Patient**		**1**	**2**	**3**	**1**	**2**	**3**	**1**	**2**	**3**	**1**	**2**	**3**	**1**	**2**	**3**	**1**	**2**	**3**	**1**	**2**	**3**	**1**	**2**	**3**	**1**	**2**	**3**	**1**	**2**	**3**
**SCM**	Dominant	−	102	95	−	13.5	10.2	−	64	81	−	64.1	26.5	−	435	581	−	11.8	13.6	−	107.0	110.1	−	−	10.4	−	10.6	8.5	−	−	−
	Nondominant	128	169	137	5.5	10.2	9.4	26	70	91	23	51	26.2	1046	876	182	18.2	16.7	16.1	112.2	105	25.7	3.4	−	63.8	8.8	7.1	8.8	−	−	−
**TRAP**	Dominant	−	91	88	−	10.9	10.8	−	77	74	−	61.5	55.3	−	454	338	−	12	10.4	−	250	113.9	−	79.5	2.5	−	13.8	11.8	−	93	−
	Nondominant	112	120	82	12	13.7	11.0	1454	98	57	59.2	63.4	53.4	1061	87	481	18.4	5.3	12.4	33.2	108.4	115.8	1	2.1	4.6	4.8	10.6	4.9	−	111	86
**ZYG**	Dominant	44	53	80	3.3	1.4	3.7	42	61	142	31	14.2	31	533	488	850	13	16.5	12.5	4.9	118.7	219.6	0.4	7.7	46.3	1.1	25.6	12.8	−	76	106
	Nondominant	53	56	114	6.3	2.1	2.7	266	57	57	7.9	15.2	28.6	1117	696	273	18.9	14.9	9.3	8.8	105.9	107.2	1.5	21.2	7.1	0.6	42.7	18.7	−	−	26
**DEL**	Dominant	−	84	68	−	11.3	13	−	62	96	−	−	54.5	−	−	140	−	−	6.7	−	121.4	103.5	−	6.8	2.6	−	7.1	11.8	−	141	109
	Nondominant	104	95	84	11.1	11.2	12.4	68	70	171	59.8	61.7	48.3	651	148	105	14.4	6.9	5.8	26.8	216.5	110	0.8	18.6	−	3.3	8.5	8	−	116	104
**BBM**	Dominant	85	81	59	12.5	16.5	16.6	787	86	71	54.2	57	57.9	777	446	54	15.7	11.9	4.1	30.5	100.7	103.7	0.3	4.3	4.3	4.1	13.3	7.3	−	127	68
	Nondominant	80	85	76	10.9	16.7	16.2	1160	53	63	56	54.4	45.1	1100	64	960	18.7	4.5	17.5	13.7	202.8	295.5	3.4	50.3	43.5	3.6	11.6	10	−	108	93
**FDI**	Dominant	123	54	46	20.2	23.4	20.4	104	111	113	47.4	51.2	56.4	175	238	261	7.5	8.7	9.1	53.7	131.0	120.8	1.4	14.1	5.8	1.7	15.7	16.6	−	116	61
	Nondominant	120	80	66	21.3	19.4	22	127	111	666	53.4	53.5	43.4	145	96	522	6.8	5.5	12.9	40.8	124.8	−	0.4	10.8	0.4	0.8	12.3	18.1	−	141	117

Muscle specific stimulation parameters in lesioned subjects.

*Abbreviations*: RMT – resting motor threshold; CSP – cortical silent period; SCM – sternocleidomastoid muscle; TRAP – trapezius muscle; ZYG – zygomaticus muscle; DEL – deltoid muscle; BBM – biceps muscle; FDI – first dorsal interosseus muscle.

**Table 4:** The muscle specific stimulation parameters of the 6 determined muscles in 15 healthy subjects are displayed. The dominant and nondominant side, as well as the difference between them, are shown above. In detail, the median and the interquartile range [in square brackets] of the dominant and nondominant side and the p-value are displayed.

**Muscle**	S**ide**	**RMT [V/m]**		**Latency [ms]**		**Amplitude [μV]**		**Distance to lateral sulcus [mm]**		**Area [mm^2^]**		**Radius [mm]**		**V50**		**Slope**		**Area under the curve**		**CSP [ms]**	
**SCM**	Dominant	112.0 [37.0]	**0.05**	11.2 [5.1]	**0.01**	94.0 [37.0]	0.10	29.2 [18.5]	1.00	756.0 [811.0]	0.26	15.5 [8.4]	0.26	103.9 [17.0]	0.22	7.6 [4.9]	0.59	10.8 [7.7]	0.94	−	−
	Nondominant	111.0 [32.0]		10.2 [3.0]		128.5 [84.0]		30.3 [16.9]		494.0 [476.0]		12.5 [5.2]		110.6 [165.7]		10.8 [53.6]		9.6 [7.2]		121.5	
**TRAP**	Dominant	85.0 [27.5]	0.28	11.7 [2.8]	0.59	130.0 [75]	0.40	54.2 [12.5]	0.78	264.0 [307.0]	**0.04**	10.1 [5.1]	**0.04**	117.7 [86.0]	0.48	6.6 [28.2]	0.72	9.6 [4.8]	**0.01**	80.5 [4.7]	0.50
	Nondominant	96.0 [29.0]		12.3 [2.9]		88.0 [44]		55.1 [8.8]		575.8 [386.3]		13.8 [4.8]		135.9 [160.7]		12.5 [17.2]		5.7 [5.6]		99.0 [49.0]	
**ZYG**	Dominant	91.0 [23.0]	0.46	11.0 [3.1]	0.48	76.0 [80.0]	0.67	23.9 [11.2]	0.76	474.0 [360.0]	0.17	12.4 [4.3]	0.11	115.5 [13.5]	0.13	11.4 [10.0]	0.53	13.1 [8.5]	0.83	111.0 [27.0]	**0.06**
	Nondominant	96.0 [20.0]		10.5 [3.8]		94.0 [36.0]		26.2 [7.6]		277.6 [371.5]		8.3 [6.2]		116.0 [18.6]		9.6 [12.9]		11.1 [8.4]		107.0 [50.0]	
**DEL**	Dominant	74.0 [23.0]	**0.02**	13.0 [1.2]	0.37	131.0 [67]	0.73	55.5 [9.7]	0.11	276.0 [300.0]	**0.09**	9.4 [4.8]	**0.08**	118.3 [175.5]	0.36	6.1 [37.3]	0.81	6.7 [5.5]	0.35	99.0 [85.0]	0.97
	Nondominant	91.0 [36.0]		12.6 [1.6]		127.0 [114.0]		54.4 [10.8]		510.0 [281.0]		12.7 [3.6]		159.2 [94.3]		10.8 [21.4]		10.4 [3.8]		110.5 [71.0]	
**BBM**	Dominant	67.0 [17.0]	**0.04**	16.2 [2.0]	0.27	94.0 [93.0]	0.53	49.9 [9.4]	0.94	398.0 [314.0]	0.59	11.2 [4.6]	0.51	104.7 [27.7]	**0.08**	9.2 [19.6]	0.88	12.6 [14.4]	0.46	88.0 [75.0]	0.59
	Nondominant	74.0 [51.0]		15.8 [1.8]		83.0 [48.0]		49.0 [11.9]		429.5 [422.7]		11.7 [5.6]		112.1 [23.3]		8.8 [9.4]		14.5 [10.4]		91.0 [56.0]	
**FDI**	Dominant	61.0 [13.0]	**0.03**	23.2 [1.9]	0.62	237.0 [237.0]	0.12	46.0 [10.2]	0.73	240.0 [402.0]	0.28	8.7 [6.3]	0.22	124.1 [22.5]	0.12	8.9 [7.3]	0.97	15.3 [15.2]	**0.06**	126.0 [37.0]	0.73
	Nondominant	70.0 [13.0]		23.4 [1.3]		492.0 [638.0]		47.3 [9.5]		354.0 [222.9]		10.6 [3.3]		117.5 [11.7]		8.6 [3.9]		18.5 [15.5]		137.0 [67]	

Muscle specific stimulation parameters in healthy subjects: dominant and non-dominant side compared.

*Abbreviations*: RMT – resting motor threshold; CSP – cortical silent period; SCM – sternocleidomastoid muscle; TRAP – trapezius muscle; ZYG – zygomaticus muscle; DEL – deltoid muscle; BBM – biceps muscle; FDI – first dorsal interosseus muscle.

## 4 Discussion

The aim of the present study was to describe the detailed spatio-functional representation of the trapezius and the sternocleidomastoid muscle within the precentral gyrus according to the homuncular organization of the motor cortex. We provided an overview of the location of the sternocleidomastoid muscle and trapezius. Our data show a dissociated representation with the sternocleidomastoid muscle lateral to the face and the trapezius around, but more superior to the hand knob. Our results are coherent with another study (Penfield W, 1950), contradicting others (Kang et al., 2011, Thompson et al., 1997). The most common TMS parameters were presented and compared with three patients with an accessory nerve lesion. RMTs were elevated on the nds when the nerve on the ds was transected. One patient exhibited an increased distance to the lateral sulcus of the sternocleidomastoid muscle, particularly on the affected ds. In cases with complete nerve transection, sternocleidomastoid muscle area on the ds was either significantly reduced or not detectable. Conversely, in the patient with nerve compression due to scar tissue, the sternocleidomastoid muscle area on the ds appeared enlarged.

### 4.1 Topography of NXI innervated muscles

We located the representation of trapezius muscle on the motor cortex around and superior to the hand knob, between the representation of the arms and the legs. In contrast to that, sternocleidomastoid muscle had been found near and overlapping the representation of a facial muscle. An important point to consider is the embryonic development and the origin of both sternocleidomastoid and trapezius muscle, which provides several possible homuncular localizations. In terms of embryogenesis, trapezius and sternocleidomastoid muscle are from a shared origin (Cho et al., 2020). Both belong to the biological developmental group of pharyngeal muscles, also known as branchial muscles (Matsuoka et al., 2005). As the other pharyngeal arches serve as the origin of muscles of the head and neck, a distinct representation around the head area must be considered. In contrast to that, the homuncular organization depends on the anatomical localization and function of muscles, a so-called somatotopic organization of movement (Roux et al., 2020). According to that, it would be also possible to find responses for trapezius and sternocleidomastoid muscle in the arm/shoulder/torso region on the precentral gyrus. It is surprising that the two muscles are represented relatively far away from each other. Our findings are consistent with the results of the aforementioned study (Penfield W, 1950) on the localization of the sternocleidomastoid muscle in the lateral motor cortex adjacent to the facial area. They thus contradict other studies (Kang et al., 2011, Thompson et al., 1997) that have localized sternocleidomastoid muscle more medially. Although the sternocleidomastoid muscle hotspot has always been found at the lateral location, there were also several positive responses further medial in the area around the trapezius localization. This raises the question of a variable representation of the sternocleidomastoid muscle on the motor cortex. From a clinical point of view, these findings provide new insights into the distinct cortical representation of the trapezius and sternocleidomastoid muscles and the cortical representation of neck and shoulder movement control. For instance, these finding might contribute to a better understanding of focal dystonias, such as cervical dystonias, and why affected muscles vary from patient to patient. Also, our findings could be a helpful base for neurofeedback therapy in neurorehabilitation, for example post-stroke in case of asymmetric head and shoulder movement or in the context of neuromodulatory therapies such as TMS.

### 4.2 Technical considerations

TMS parameters in general vary strongly between subjects and within one subject over time. There are no correlations to sex but for age and strong correlations between siblings for the RMT, for example (Saisanen et al., 2008, Wassermann, 2002). To measure only fifteen subjects once could result in imprecise RMT values. Other factors influencing the precision of the RMT are a superficial or a deeper position of the muscle in the body, for example the sternocleidomastoid muscle being covered by the platysma. Also, turning the head should be avoided due to a resulting displacement of the electrodes. These points could be avoided by using needle electrodes, but it is obviously more invasive. An imprecise determination of the RMT of a muscle also results in a limited comparability of the muscles in the area, its representation area and the amplitude. Latency depends in addition on the height of the subject, apart from correlating with the distance of the respective muscle from the motor cortex. Concerning the variability in the amplitudes of the MEPs across muscles, variations in the density of the cortical representation and the corticospinal connectivity could influence the results. Muscles like the first dorsal interosseus usually show particularly high MEP amplitudes due to their strong monosynaptic corticospinal connections and dense cortical representation.

An important point that needs to be discussed in terms of feasibility is the location of the sternocleidomastoid muscle on the motor cortex, which is partly covered by the temporal muscle. A major problem when stimulating the sternocleidomastoid muscle, but also the zygomatic major, is the simultaneous stimulation of the temporalis muscle and thus the triggering of a rapid and sometimes strong jaw closure, which in turn leads to a movement in the tissue around the jaw, which includes the zygomatic major and sternocleidomastoid muscle bilaterally. This could be misinterpreted as a MEP by the EMG. These false-positive spikes often have a shorter latency than the mean latency of true responses and do not have the typical shape.

### 4.3 Feasibility of nTMS in patients with NXI lesions

In patients with a severe lesion of the accessory nerve, there is a high probability that the affected muscles cannot be stimulated. The probability increases even further with time after the lesion because the muscle will atrophy.

Another important point is the duration of the mapping. At the beginning of the protocol implementation, the examiner needed five hours to carry it out. Any pathologies, such as those present in the patients, could prolong the session even further, so that fatigue of both the subjects and the examiner could potentially influence the results. For future studies, the protocol should be shortened to the essential parameters.

One study determined the CSP of sternocleidomastoid muscle and trapezius computer-aided (Nilsson et al., 1997). They induced a tension in the sternocleidomastoid muscle by letting their subjects lift their heads off a headrest against pressure − this setup could not be applicable in all patients because of nerve palsy. For trapezius, lifting the shoulders produced a CSP which was about twice as long as the one in this study (194.2 ± 28.8 vs. 91.9 ± 32.3 ms). Another study determined the CSP of trapezius as 131.0 ± 6.3 (Menon et al., 2018). A main reason could be that a computer-aided determination is not standard in every lab. In our study, the CSP was quantified manually by measuring the silent part of the EMG, where the endpoint of this period can vary through subjective definition of where the EMG gets too curvy. Others defined the endpoint of the CSP as the point where the EMG reached 50 % of the previous background activity (Werhahn et al., 1995). Also, the CSP changes with the stimulus intensity, which depends on the RMT and the level of suprathreshold stimulation.

### 4.4 Outlook

This study compares the most common TMS parameters for the sternocleidomastoid muscle and trapezius as well as four other functionally and spatially related muscles in fifteen healthy subjects and three patients with an accessory nerve lesion. The results could serve as a basis for future TMS studies in which cortical plasticity after lesions of the accessory nerve could be investigated in more detail. After describing the area and location of the sternocleidomastoid muscle and trapezius representation on the precentral gyrus, it would be interesting to investigate whether the area changes in size or location, for example, over time depending on the clinical course. It could be assumed that the RMT increases or the amplitude decreases in muscles with damaged nerves. Differences in the parameters between the hemispheres could also be a possible finding. Conclusions from this type of study could be used to predict outcomes or decide on treatment and timing after peripheral nerve lesions.

### 4.5 Limitations

Due to the time-consuming mapping protocol, our sample size was relatively small resulting in a limited generalizability. Mapping was performed by two well trained novice examiners. Concerning interoperator variability, nTMS is described as a reliable mapping tool with low interoperator variability and only small differences between expert and novice examiners (Zdunczyk et al., 2013).

## 5 Conclusion

It serves as a pilot study for subsequent research to investigate pathological pathways after accessory nerve injury. Further nTMS studies and a larger number of subjects are needed to find relationships between factors of cortical reorganization following nerve injury and time or technique of surgical vs. non-surgical repair. nTMS after an (accessory) nerve injury has the potential to improve the accuracy of an early prognosis or to provide a structured decision-making process for the management of the injury.

**Author contributions**

J. Reinsch was responsible for drafting the manuscript, conducted the majority of the measurements, and performed data analysis. M. Denker contributed to a smaller portion of the measurements and was involved in the conceptualization of the study together with J. Reinsch and N.F. Dengler. M. Engelhardt instructed J. Reinsch and M. Denker in the measurement technique and supported the development of the measurement protocol. D. Huscher provided statistical expertise and assisted with the data analysis. P. Vajkoczy supervised the department and supported the project at the institutional level. T. Picht contributed to the conceptual design of the study and assisted in the development of the measurement protocol. N.F. Dengler was primarily responsible for the study concept, performed the surgical procedures, and supported both the manuscript preparation and the structural design of the study.

## Declaration of competing interest

The authors declare that they have no known competing financial interests or personal relationships that could have appeared to influence the work reported in this paper.
